# Glial suppression and post-traumatic stress disorder: A cross-sectional study of 1,520 world trade center responders

**DOI:** 10.1016/j.bbih.2023.100631

**Published:** 2023-05-13

**Authors:** Ginny Natale, Minos Kritikos, Pei-Fen Kuan, Melissa A. Carr, Xiaohua Yang, Yuan Yang, Roman Kotov, Evelyn J. Bromet, Sean A.P. Clouston, Benjamin J. Luft

**Affiliations:** a Program in Public Health and Department of Family, Population, and Preventive Medicine, Renaissance School of Medicine at Stony Brook University, Stony Brook, NY, USA, 11794; b Department of Applied Mathematics, Stony Brook University, Stony Brook, NY, USA, 11794; c Stony Brook World Trade Center Wellness Program, Renaissance School of Medicine at Stony Brook University, Stony Brook, NY, USA, 11725; d Department of Medicine, Stony Brook University, Stony Brook, NY, USA, 11794; e Department of Psychiatry, Stony Brook University, Stony Brook, NY, USA, 11794

**Keywords:** Post-traumatic stress disorder, Glial fibrillary acidic protein, Neuroinflammation, World trade center, Disasters

## Abstract

**Background:**

Chronically re-experiencing the memory of a traumatic event might cause a glial response. This study examined whether glial activation would be associated with PTSD in a study of responders present after the 9/11 World Trade Center attacks without comorbid cerebrovascular disease.

**Methods:**

Plasma was retrieved from 1,520 WTC responders and stored for a cross-sectional sample of responders of varying levels of exposure and PTSD. Plasma levels (pg/ml) of glial fibrillary acidic protein (GFAP) were assayed. Because stroke and other cerebrovascular diseases cause distributional shifts in GFAP levels, multivariable-adjusted finite mixture models analyzed GFAP distributions in responders with and without possible cerebrovascular disease.

**Results:**

Responders were aged 56.3 years and primarily male; 11.07% (n = 154) had chronic PTSD. Older age was associated with increased GFAP, whereas higher body mass was associated with decreased GFAP. Multivariable-adjusted finite mixture models revealed that severe re-experiencing trauma from 9/11 was associated with lower GFAP (B = −0.558, p = 0.003).

**Conclusion:**

This study presents evidence of reduced plasma GFAP levels among WTC responders with PTSD. Results suggest re-experiencing traumatic events might cause glial suppression.

## 1 Introduction

Glial cells have an array of functions, including interfacing with oligodendrocytes to maintain homeostasis and activate in response to local neuroinflammation (Quincozes-Santos et al., 2021). When glial cells react to stress, they emit a neuroinflammatory protein known as glial fibrillary acidic protein (GFAP), suggesting that GFAP might be a putative biomarker of pro-glial activation (Abdelhak et al., 2022). Histological studies have further found that GFAP protein are expressed by reactive glia and have been found to match levels of neuroinflammation (Brunkhorst et al., 2010; Foerch et al., 2006, 2012). While the role of glial cells in maintaining brain homeostasis is well understood, it is unclear whether re-experiencing traumatic events can independently cause a glial response.

Changes in glial activation have been identified in post-traumatic stress disorder (PTSD) (McEwen and Rasgon, 2018). Chronic PTSD occurs when World Trade Center (WTC) responders with chronic PTSD repeatedly re-experience the traumatic event (Bromet et al., 2016), which may cause a glial response, as PTSD has been linked to neuroinflammation (Sun et al., 2021). A study of WTC responders with chronic PTSD found that monocytes were being chronically activated, with sequencing results suggesting these monocytes, which can act as cerebral macrophages, were primed to respond to viral invaders in the brains of responders with PTSD (Kuan et al., 2019), further suggesting a neuroinflammatory environment that may elicit glial activation. Another study of Veterans with chronic PTSD found evidence of neuroimmune suppression immediately after trauma exposure in early adulthood (Bhatt et al., 2020). Other studies report that PTSD is associated with peripheral inflammation, acting as a catalyst to a neuroinflammatory cascade whereby astroglia express an array of anti-inflammatory proteins (Kettenmann et al., 2011; Villegas-Llerena et al., 2016) and tamp down other inflammatory responses. Some studies find evidence of lower pro-inflammatory cytokine levels in people with PTSD in blood-based studies (McCanlies et al., 2011; Plantinga et al., 2013; Sondergaard et al., 2004) and cerebrospinal fluid studies (Baker et al., 2001; Bonne et al., 2011), though findings are mixed. One PET study of PTSD symptomatology reported the presence of heightened glial activation among those with more severe PTSD symptoms (Deri et al., 2021). Lastly, a meta-analysis said that adults with PTSD had elevated levels of circulating peripheral immune biomarkers compared to healthy controls (Passos et al., 2015).

It remains unclear as to whether there is any glial reaction to PTSD and, if so, whether that reaction would cause heightened or suppressed GFAP. Measuring plasma GFAP is an affordable, quick, efficient, and low-risk venipuncture alternative to neuroimaging studies. We hypothesized that chronic PTSD would be associated with GFAP levels. We further examined the relationships between GFAP distributions associated with PTSD severity; and GFAP volume related to re-experiencing the traumatic event(s).

## 2 Materials and methods

### 2.1 Setting

The thousands of workers and volunteers who participated in the rescue and recovery efforts following the attacks on the World Trade Center (WTC) on September 11th, 2001 (9/11), were exposed to an extraordinary array of psychological traumas (Neria et al., 2011) and toxic exposures (Lioy and Georgopoulos, 2006). The current analysis focuses on responders participating in the World Trade Center Aging Study at Stony Brook University. As responders age, research shows that responders are experiencing an elevated risk of symptoms consistent with rapid neurological aging and the presence of neurodegenerative disease. As a result, they have long shared a high risk of physical and mental health disorders, including a chronically elevated risk of post-traumatic stress disorder (PTSD) (Bromet et al., 2016). The severity of WTC exposure and the presence of chronic PTSD are the two most salient risk factors (Clouston et al., 2022).

We assessed GFAP in the plasma of 1,529 WTC responders with valid diagnostic information of PTSD and WTC exposure variables who were eligible for this study. The research team linked GFAP protein distribution to existing WTC monitoring records that included PTSD symptoms obtained and measured serially since baseline. We excluded five responders who reported any head injury suffered while on-site and four responders with missing information on demographics, leaving a final analytic sample of N = 1,520 responders.

### 2.2 Glial fibrillary acidic protein (GFAP) serology collection and assay techniques

GFAP volumes in the plasma (possible detection range 0–4000 pg/mL) were analyzed using Simoa ®, an ultra-sensitive analyzer that is a bead-based enzyme-linked immunosorbent assay (ELISA). Whole blood samples from responders were collected in K2-EDTA blood collection tubes, placed on ice, and then centrifuged at 2,000 g for 15 min at 4 °C. Plasma samples were separated and placed into polyethylene tubes before being stored at −80 °C until shipping. Plasma samples to be assayed at Quanterix™ laboratories were transported on dry ice. Samples were auto diluted at 4X. The average coefficients of variation (intraplate and inter-plate) values were below 10%. GFAP volumes were calculated using the composite approach. Results reported represent averaged results across duplicate arrays, and all values were expressed in pg/ml. The limit of detection (LOD) was 0.211 pg/mL (0.108–0.284 pg/mL) and was calculated as 2.5 standard deviations from the mean background signal read back on each calibration curve over one reagent lot per instrument. The lower limit of quantification (LLOQ) was 0.686 pg/mL and was measured in triplicate measurements using a serially diluted calibrator and was read back on the calibration curve over one reagent lot on a single instrument (five runs). No participants had plasma biomarker levels below the lower limit of quantification (∼0.01 pg/mL).

### 2.3 Post-traumatic stress disorder (PTSD)

PTSD symptoms were assessed at each monitoring visit using the PTSD checklist-specific trauma version tailored to the WTC disaster (PCL-17 trauma-specific version) (Wilkins et al., 2011). Individuals rated the extent to which they were bothered by PTSD symptoms relating to WTC exposures in the past month on a scale from 1 (not at all) to 5 (extremely). Items were summed into an overall severity score and also were summed within four distinct PTSD symptom dimensions consistent with four-factor models of PTSD dimensionality (King et al., 1998): re-experiencing the event (e.g., flashbacks/nightmares), effortful avoidance (e.g., actively avoiding reminders), emotional numbing (e.g., emotionally distancing from life), and hyperarousal (e.g., being ever aware and on edge) (Cronbach's α = 0.96). Current PTSD status was determined using the PCL using a cutoff of ≥44 to indicate probable PTSD for descriptive purposes. Since different domains of PTSD often indicate different outcomes, we evaluated the importance of distinct domains, and found that relying on re-experiencing symptoms improved prediction thereby justifying our reliance on this subset of symptoms in analyses.

### 2.4 Covariates

*Demographic variables* included age (in years), female sex, race/ethnicity, and educational attainment. Race/ethnicity included four categories: Non-Hispanic White, Black, Other/Mixed, and Hispanic ethnicity. Education (%) was categorized into: High school or Less, Some college (<2 years), and a 4-year university degree.

*Physical Health variables included*: Smoking status (e.g., % of current, never, former smoker); Body Mass Index (BMI) in kg/m^2^; Height (cm); history of ever having received a diagnosis of Hypertension (yes/no), Diabetes (yes/no), or Stroke (yes/no) prior to the screening visit.

The literature has also reported that hemorrhagic stroke can cause a nearly 20-fold increase in GFAP levels within 4 hours of the event (Katsanos et al., 2017) while other conditions do not cause such enormous increases (Schiff et al., 2012). Since strokes are underdiagnosed and the goal of the study was to determine glial distribution under normal cerebral operations, it was imperative that we consider the confounding presence of strokes and other potentially undiagnosed cerebrovascular conditions in a study of GFAP among individuals with and without chronic PTSD.

### 2.5 World trade center exposures

*WTC exposure variables included* Non-Traditional Responder, Exposure Duration; Dust Cloud Exposure; and No Supervisory Work. *Non-Traditional Responders* (%) present on the WTC site included anyone who was not trained to be a responder to accidents and trauma, for example, electricians, engineers, bus drivers, good Samaritans, and volunteers. *Exposure Duration* was measured in the number of weeks a responder was on the WTC site beginning November 9, 2001. *Dust Cloud Exposure* measured the % of the time a responder was under a dust cloud from the WTC attacks. *No Supervisory Work* (%) measured the number of responders on the ground working tasks compared to Officers or others who primarily did supervisory work away from the most intense exposures.

### 2.6 Statistical analyses

Descriptive sample statistics (means and standard deviations or frequencies and percentages) were calculated for the entire sample and within subgroup comparisons. We expected that GFAP would stratify from prior literature into two subclasses, indicating a Normal Class of neurological operations versus a Pathological Class due to the presence of brain trauma or stroke (i.e., cerebrovascular disease). However, GFAP cutoffs are not well validated and may differ depending on age, gender, race/ethnicity, and even blood volume. A multivariable-adjusted finite mixture modeling (below) determined two classes to the GFAP distribution, with one class represented by a normal distribution of GFAP and another class characterized by the highest-high GFAP levels associated with brain injury or stroke. Finite mixture modeling allows us to model the presence of classes, including, for example, a Pathological Class, while jointly modeling correlates of GFAP distribution within either subclass as necessary. We assumed that GFAP distributions in the Normal Class might be related systematically to several variables that follow a Gaussian distribution. In the Pathological Class, however, we assumed that GFAP distributions would follow a continuous Log-Gamma distribution as it is common in indicators that are biased monotonically by a disease. Sensitivity analyses examined whether to use more classes; the two-class model performed the best (BIC = 14,050 *versus* 14,977 using a single class solution) and is reported here (Appendix Table 1). A quantile-quantile plot upheld the 10.13039/100014230Gaussian distributional assumptions. Supporting this decision, we found that the skew and kurtosis were within normal parameters (s = 0.46, k = 2.59). The Pathological Class was non-Gaussian and highly skewed, so we used the Gamma distribution to measure the serologic protein volume. These two classes were derived from the model directly for bivariable comparisons and to describe these classes. For descriptive purposes, class membership was estimated using posterior predicted values; a cutoff value ≥ 0.10 identifying participants in the Pathological Class. This value appeared to differentiate the highest-high GFAP levels from the low to low-high GFAP levels in distributional analyses. Sensitivity analyses determined whether the highest-high GFAP cutoffs were a primary driver of reported results to address potential bias (Appendix Table 2). We further examined symptom-specific predictors of PTSD and depression on the distribution volume of GFAP using OLS regression (Appendix Table 3).

**Table 1:** Descriptive characteristics for the sample and separated by the Normal versus Pathological Classes as determined using finite mixture modeling, World Trade Center Aging Study (N = 1520).

	Whole Sample		Normal Class		Pathological Class*		P value
	M	SD	M	SD	M	SD	
GFAP, pg/mL	77.37	42.00	68.32	23.47	174.99	66.79	<0.001
Age	56.32	7.95	55.44	7.17	65.80	9.55	<0.001
Post-Traumatic Stress, Re-experiencing Symptoms	0.27	0.37	0.26	0.36	0.34	0.45	0.027
Exposure Duration, weeks	26.34	18.98	26.37	18.93	25.94	19.59	0.925
Body mass, kg/m^2^	31.07	5.27	31.24	5.26	29.16	5.00	<0.001
Height, cm	177.66	7.87	177.83	7.89	175.86	7.34	0.003

	%		%		%		P value
Female sex,	28.42		26.96		44.19		0.344
Race/Ethnicity,							
White	88.55		88.57		88.37		0.071
Black	2.89		2.59		6.2		
Other	1.97		2.01		1.55		
Hispanic	6.58		6.83		3.88		
Educational Attainment,							
High School or Less	24.74		24.66		25.58		0.400
Some College	47.11		47.59		41.86		
University Degree	28.16		27.75		32.56		
Smoking Status,							
Never Smoker	62.7		63.12		58.14		0.211
Current Smoker	4.87		5.03		3.10		
Former Smoker	32.43		31.85		38.76		
Hypertension,	39.34		38.03		53.49		0.001
Diabetes,	12.57		12.44		13.95		0.619
Stroke,	2.30		1.44		11.63		<0.001
Non-Traditional Responder	28.42		26.96		44.19		<0.001
Dust Cloud Exposure,	17.96		18.04		17.05		0.779
No Supervisory Work,	85.33		86.27		75.19		0.001

**Note:** P-values reported from Welch's *t*-test using an assumption of unequal variance or from ꭓ^2^ tests as appropriate across the Whole sample, Normal and Pathological Classes. *The Pathological Class contains WTC responders whose finite mixture model results for indicated that participants' GFAP levels were indicative of having had a stroke or other unspecified cerebral trauma (predicted probability ≥0.10).

Abbreviations: kg, kilograms; m, meter; cm, centimeters; pg, picograms; mL, milliliter.

**Table 2:** Characteristics of responders’ glial fibrillary acidic protein levels in the Normal Class, stratified by probable post-traumatic stress disorder status. World Trade Center Aging Proteins Study (N = 1520).

	Normal Class				P-Value
	No Post-Traumatic Stress Disorder		Probable Post-Traumatic Stress Disorder		
	M	SD	M	SD	
Glial Fibrillary Acidic Protein, pg/ml	68.88	23.39	63.80	23.64	0.005
Age, years	55.37	7.18	55.96	7.13	0.430
Exposure Duration, weeks	26.09	18.77	28.67	20.08	0.144
Body mass, kg/m2	31.15	5.21	31.96	5.60	0.063
Height, cm	177.79	7.91	178.13	7.75	0.503

	%		%		P-Value
Female sex (vs. Male)	24.82		44.16		0.701
Race/Ethnicity					
White	88.68		87.66		0.212
Black	2.83		0.65		
Other	1.86		3.25		
Hispanic	6.63		8.44		
Educational Attainment					
High School or Less	24.17		28.57		0.108
Some College	47.21		50.65		
University Degree	28.62		20.78		
Smoking Status					
Never Smoker	64.19		54.55		<0.001
Former Smoker	31.69		33.12		
Current Smoker	4.12		12.34		
Hypertension	38.08		37.66		0.921
Diabetes	11.64		18.83		0.011
Stroke	1.05		4.55		0.001
Non-Traditional Responder	24.82		44.16		<0.001
Dust Cloud Exposure	17.14		25.32		0.013
Non-Supervisory Work	86.10		87.66		0.594

**Note:** P-values reported from Welch's *t*-test using an assumption of unequal variance or from χ^2^ tests as appropriate.

Abbreviations: kg, kilograms; m, meter; cm, centimeters; pg, picograms; mL, milliliter.

**Table 3:** Multivariable-adjusted finite mixture model showing predictors of the distribution volume of glial fibrillary acidic protein in responders determined to have normal glial fibrillary acidic protein levels.

	Model 1			Model 2		
	B	SE	P	B	SE	P
Post-Traumatic Stress Disorder, Symptoms	−0.753	0.192	<0.001	−0.558	0.187	0.003
Age, years	1.070	0.115	<0.001	1.134	0.113	<0.001
Female	2.033	4.552	0.655	−1.118	4.032	0.782
Height, cm				−0.158	0.091	0.084
Body Mass, kg/m^2^				−1.050	0.120	<0.001
Exposure Duration, Ln-Weeks				−0.869	1.281	0.498
Dust Cloud				−2.330	1.571	0.138
No Supervisory Work				−2.253	1.902	0.236

**Note:** Finite mixture models (N = 1520) allow us to jointly examine differences within Normal versus Pathological Classes and for predictors of class membership. However, only results for the distribution levels for GFAP in the normal Class are shown. Finite mixture models adjusted for demographics (gender, race/ethnicity) in all models for both class membership and distribution volumes within each class. In Model 2, we adjusted class membership analyses to include a history of stroke, hypertension, diabetes, smoking status, height, and body mass, non-traditional responder status, and WTC toxic exposures.

AbbreviationsB, regression coefficient; SE, standard error; P, p-value; kg, kilograms; m, meter; Ln, natural logarithm.

### 2.7 Sensitivity analyses

Sensitivity analyses examined any changes to conclusions when modifying distributional assumptions. Analyses were also stratified to examine whether results differed across officer status and burden of PTSD. Analyses further examined the role of early enrollment by excluding individuals enrolled after 2008. Because treatment may provide a mechanism through which PTSD affects neuroinflammation, analyses examined whether medication treatment for PTSD mediated results. Because glial activation may differ in its impact on responders with dementia, we further examined whether excluding those responders with dementia changed results. Finally, prior work has found that untrained responders who worked in construction or volunteer positions helping after November 9, 2001 are often more strongly affected by their experiences (Luft et al., 2012), so we stratified results within the normal class by occupational (trained/untrained) status to determine whether associations were larger in that group. Results in stratified analyses were compared using Welch's t-tests to compare beta coefficients from seemingly unrelated regressions.

All analyses were conducted using Stata 17/MP [StataCorp].

#### 2.7.1 Ethics

The present study received approval from the Stony Brook Institutional Review Board (IRB, CORIHS #604113), and the experiment protocol involving humans was conducted in accordance with the Declaration of Helsinki. All participants provided written informed consent.

## 3 Results

### 3.1 Sample characteristics

Consistent with the interpretation that the Pathological Class had responders with cerebrovascular disease; 11.63% of responders in the Pathological Class had a history of diagnosed stroke compared to 1.44% of those in the Normal Class (relative risk = 8.08, P < 0.001). Descriptive characteristics for the sample and for the two subclasses defined by 1) responders in the Normal Class (without presumed cerebrovascular disease) and 2) in the Pathological Class (with model-estimated probability >0.50 that GFAP levels indicate a cerebrovascular disease) are shown in Table 1. Mean GFAP concentrations differed significantly between the Normal versus Pathological Classes of responders and the whole sample (Table 1). Theveragee age of responders was 56 years (p < 0.001) but was younger in the Normal Class (aged 55 years on average) and older in the Pathological Class (aged 66 years on average) (Table 1). The sample was predominantly non-Hispanic White (88.6%) compared to responders with Other race/ethnicity (1.97%), Hispanic (6.6%), and Black (2.9%). The sample of responders varied significantly (p < 0.001) between the whole sample, the Normal Class, and the potentially Pathological Class in BMI, height, cardiovascular disease, and self-reported history of stroke. Exposure duration (weeks) and 9/11 dust cloud exposure were similar across all responders in the sample (p = 0.925 and 0.779 respectively). Responders with no supervisory work and non-traditional responders varied between classes, such that the Pathological Class had lower exposure on the ground site on 9/11, were more than a decade older on average, were more likely to have worked as supervisors at 9/11 consistent with their older age, and had a higher proportion of non-traditional responders. Smoking history, higher educational attainment, female sex, and race/ethnicity did not vary between classes.

**Fig. 1:**
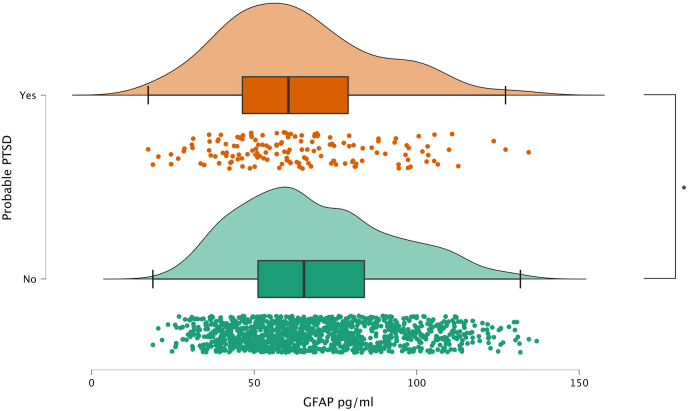
Raincloud plots showing data distribution (cloud), jittered raw data (rain), and central tendency boxplots for glial fibrillary acidic protein (GFAP) [pg/ml] as stratified by post-traumatic stress disorder classification (PTSD) groups from Table 2 in responders classified as belonging to the normal class. **Note**: Asterisk * denotes a significantly different pairwise comparison using a Welch's T-test (p = 0.005). The results here exclude responders with estimates of the probability of pathological class membership ≥0.10 consistent with Table 1.

**Fig. 2:**
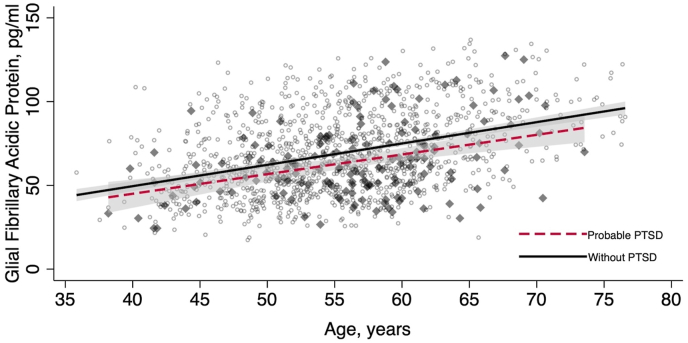
Regression models showing the relationship between age and glial fibrillary acidic protein volumes for responders with and without post-traumatic stress disorder in responders lacking evidence of glial pathology. Results show expectations derived from finite mixture models for responders in the normal class. Results for responders with probable PTSD are shown using a dashed line (with 95% confidence interval) and dark gray dots in the scatterplot; results for responders without probable PTSD are shown using a solid black line (with 95% confidence interval) and unfilled gray dots in the scatterplot.

WTC responders varied significantly by GFAP concentrations, with PTSD-affected responders having significantly lower GFAP levels than the non-PTSD group (Fig. 1). Table 2 shows the subsample group's characteristics of responders with no cerebrovascular diseases, stratified by PTSD status. Mean GFAP values were higher among responders in the Normal Class with no PTSD symptoms (68.88 ± 23.39 pg/mL, N = 1237) than those with PTSD (63.80 ± 23.64 pg/mL, N = 154) (Table 2). Fig. 2 shows a scatter plot showing the glial fibrillary acidic protein distribution by age, with the best fitting line shows the expected elevation in glial fibrillary acidic protein by age in the Normal Class.

Multivariable-adjusted analyses derived from finite mixture models examining the WTC responders’ distribution volume of glial fibrillary acidic protein in the Normal Class are shown in Table 3. Model 1, finite mixture models adjusted for demographics (gender, race/ethnicity) in all models for both class membership and distribution volumes within each class. In Model 2, we adjusted class membership analyses to include a clinical history of stroke, hypertension, diabetes, smoking status, height and body mass, occupation and training level, and WTC toxic exposures. Model 1 reported that increased symptoms of PTSD was associated with reduced GFAP levels in the Normal Class. In the multivariable-adjusted model (Model 2), PTSD symptom severity was still associated with decreased glial activation, but not significant after adjusting for body mass and height. Older age was significantly associated with elevated GFAP volumes (p < 0.001) among those with and those without PTSD, but responders with greater BMI had lower GFAP levels (p < 0.001). Additionally, bivariate, and demographically adjusted models showed that PTSD was not significantly associated with increased odds of Pathological Class membership (B = 0.048, SE = 0.035, P = 0.163).

### 3.2 Sensitivity analyses

First, we examined whether distribution assumptions changed results; however, while differences in assumptions did change the overall number of individuals in the abnormal GFAP class, we did not find that results changed expectations in the Normal Class. Follow-up analyses stratifying by trained versus untrained responder status suggested that lower GFAP levels associated with PTSD symptomatology were somewhat larger in untrained WTC responders (difference = −2.17, SE = 0.44, P < 0.001), though other associations remained similar between these groups. Next, we examined if there were differences in associations between PTSD and GFAP in the Normal Class when different class-related cutoffs were used. Using a cutoff of 0.05 rather than 0.1 resulted in 16 fewer responders being classed as having a possible stroke, while using a cutoff of 0.2 resulted in 26 additional responders having a possible stroke. The direction and size of the results showed that individuals with PTSD had lower GFAP levels (difference = −3.7 pg/mL, and −4.3 for cutoffs at 0.05 and 0.2, respectively. We also examined the potential benefit of increasing the number of possible classes from two, as reported here, to one, three and four (Supplemental Table S1 for model fit statistics). Briefly, we found that the model for 4 classes did not converge, while the model using three groups converged but did not improve fit and identified a small class of individuals with relatively low GFAP. Since hypogliosis is not a known condition, we felt that this suggested that the three-class solution was sub-optimal. We stratified analyses by occupation type, and examined the utility of adjusting for PTSD treatment but found no difference in results. Next, we examined whether an earlier or later date of enrollment in the parent monitoring program might result in differences in outcomes by stratifying analyses by year of enrollment but found that such stratification did not substantively change results and that results remained significant in those who were enrolled before 2009 (P = 0.012). Finally, though not a central goal of this study we examined whether results differed when individuals with a history of mild cognitive impairment or dementia were excluded and found that results were similar and remained statistically significant (p < 0.001).

## 4 Conclusions

PTSD is a common condition among many individuals exposed to traumatic life events, and in WTC responders, chronic PTSD appears to be associated with poorer brain health. In this study, we found that more severe symptoms of PTSD were inversely associated with lower GFAP volumes in adults without suspected stroke pathology, which contributes to our understanding of neuroimmunological mechanisms in chronic PTSD. Decreases in glial activation could indicate that the brain's immune system is not achieving neuroimmunological homeostasis. Results are consistent with an independent postmortem investigation reported neuroimmune dampening as measured using positron emission tomography imaging and the expression of microglia-associated genes. Results support the hypothesis that chronic PTSD is associated with glial tamping and may indicate decreased ability to mount a cerebral immunologic defense.

Chronic activation of neuroimmune response following a reentrant stressful memory may be problematic, as glial activation supports regular brain operation and controls synaptic plasticity (Bellver-Landete et al., 2019; Parkhurst et al., 2013). Some researchers, however, report a consistent over-expression of neuroinflammatory markers in people with PTSD (Passos et al., 2015). Yet, while PTSD is consistently diagnosed across populations, results between studies of PTSD using different measures of neuroimmune functioning are inconsistent. For example, studies of WTC responders (Rosen et al., 2017) and veterans both have reported increased levels of CRP in those with PTSD (Bhatt et al., 2020). Additionally, studies of WTC responders have reported overactivation of inflammatory mechanisms in cell subpopulations focused on monocytes (Kuan et al., 2019) which were linked to neural response to biotic and stressful exposures (Kuan et al., 2021). Other studies of WTC responders report upregulation of glia (measured using F18-FEPPA) in responders with more PTSD symptoms (Deri et al., 2021). In contrast, studies of Veterans report heightened levels of CRP accompanied by glial tamping as measured using C11-PBR28 in PTSD (Bhatt et al., 2020).

Perceived inconsistencies in the results above may be due to the redundant, phase-specific nature of the neuroimmune system: the neuroimmune system relies on glia as a first line defense but can also recruit monocytes from the blood to act as cerebral macrophages as a second line of defense, and both cells have pro- and anti-inflammatory phases (Spiteri et al., 2022). For example, reactive glia and macrophages all express cytokines, chemokines, growth factors, and other proteins to attenuate or avoid brain damage caused by trauma (Nordengen et al., 2019). Yet, macrophages do not express translocator protein (TSPO) to the same extent as glial cells and TSPO ligands seem also to differ in measurement sensitivity with F18-FEPPA specifically targeting M2 glial cells (Zammit et al., 2020), while C11-PBR28 may target macrophages irrespective of phase (Chen et al., 2021). In contrast, GFAP levels are consistently expressed by glia but inconsistently expressed by macrophages – with M2 macrophages upregulating GFAP expression while M1 macrophages downregulating GFAP expression (Haan et al., 2015). Thus, one conclusion from these seemingly inconsistent results is that PTSD elicits a robust macrophagic response (both pro- [M1] and anti-inflammatory [M2]) but also elicits a muted or tamped glial response in comparison, ultimately resulting in reductions in GFAP.

While changes in GFAP seen here are believed to reference changes in glial activation, GFAP must not only be expressed by glia but must also avoid binding to the brain and ultimately be shed into the blood. GFAP in older adults with evidence of high cerebral amyloid burden, TBI, or stroke are consistently shown to be increased (Abdelhak et al., 2022). Yet, GFAP levels could be significantly reduced in individuals with other neuropathologies specifically because GFAP might be binding directly to the cerebrum thereby avoiding being sloughed into the blood (Pereira et al., 2021). A prior study of WTC responders with PTSD and mild cognitive impairment found downregulation of markers of inter-neuronal damage (e.g., , Neurocan, and Brevican) among responders with PTSD that were thought to be downregulated because of their increased cerebral absorption (Kuan et al., 2020; Waszczuk et al., 2023). Similarly, studies have noted that PTSD is associated with changes to glucocorticoid activation via activation of the FKBP5 gene (Kuan et al., 2017, 2019), a result that could downregulate glia (Nichols et al., 2005). These results may, therefore, reflect the possibility that people with PTSD are developing brain injuries that are relatively unique in terms of how and where levels of GFAP are expressed or absorbed.

### 4.1 Clinical implications

Concerning therapeutics, the development of novel treatment strategies based on a deeper understanding of biological mechanisms underlying PTSD, such as inflammatory pathology, is of particular significance. Hori and Kim (2019) proposed that elevated pro-inflammatory markers are useful in classifying (stress-related) psychiatric disorders with PTSD, perhaps constituting a model case in which heightened inflammation resides at the crossroads where peripheral inflammation meets neuroinflammation. However, increased neuroinflammation is not observed in all patients with PTSD, appears to depend somewhat on the measure of inflammation used in the study and is not a unique feature of PTSD compared to other neuropsychiatric disorders. Regarding therapeutic intervention, results may suggest that anti-inflammatory medications could have variable impact on individuals with PTSD if they act on glia as compared to macrophages.

### 4.2 Limitations

Though being the first cross-sectional study to describe the distribution of GFAP in individuals with PTSD or WTC responders in general, our study has several limitations that should be noted when trying to understand its conclusions. First, this study sought to understand a putative biomarker for brain activity. Previous work in this area has used the gold standard of neuroimaging studies to consider this research topic on stress-related psychiatric disorders. The present study cannot localize the impact of GFAP within the brain, nor can it examine the impact of neuroinflammation within the brain. Yet, this study incorporated information on a scale that is impossible for neuroimaging studies and therefore allowed us to examine the potential for other covariates to modulate the results of this study. Furthermore, while neuroimaging studies are limited in investigating individuals with high body mass, anxiety and/or claustrophobia, and dangerous metal implants, this study could specifically examine those individuals. Our sample was predominantly male, and nearly all responders in the cohort worked in first responder occupations (e.g., police, firefighter, emergency medical, etc.), or in construction-based occupations in 2001 when they were traumatically exposed. The exposures on-site at the WTC were relatively unique, and while we did not see a large exposure effect, we cannot rule out the potential that other exposures than those reported here may change the brain's immune response. Yet, while these factors may reduce the generalizability of effects, the experience and symptoms of PTSD are like those in other cohorts and due to other exposures and, therefore, the generalizability of the distal effects of PTSD are generally thought to be highly generalizable to PTSD in other groups in the general population whose PTSD does not emerge in conjunction with unique exposures seen of combat like concussive blasts.

### 4.3 Conclusion

Immunological dysregulation in PTSD may include the loss or suppression of neuroprotective microglia, resulting in decreased GFAP volumes associated with increased PTSD symptom severity. Glial activation can be both helpful and harmful, thus the interpretation of lower GFAP volumes in PTSD remains unclear at this time. For example, the presence of heightened GFAP following a stroke likely reflects activation of helpful glia seeking to repair damage caused by the stroke – in a sample without stroke, these results may operate quite differently and indicate that chronic suppression of anti-inflammatory glia could result in poorer brain maintenance and increased risk of cerebral atrophy in the long-term. As such, studies investigating how best to support regular maintenance activities in the neuroimmune system of individuals with PTSD may be warranted.

## Disclosure statement

The authors declare that they have no competing interests.

## Ethics approval

IRB approval was obtained before conducting this study from Stony Brook University.

## Consent for publication

Subjects have consented to the publication of the data.

## Author contributions

S.C. conceived and designed the methods, and G.N. performed the literature analysis and drafted the article. S.C. and M.K. designed data visualization figures and Tables and gave critical comments on the manuscript. All authors approved the final version of this manuscript.

## Declaration of competing interest

This is an epidemiological study examining biomarkers associated with posttraumatic stress disorder. The authors have no conflicts of interest to disclose.

## Data Availability

Data will be made available on request.
