# Supplementary material for: Glial suppression and post-traumatic stress disorder: A cross-sectional study of 1,520 world trade center responders
Source: Brain Behav Immun Health. 2023 May 13;30:100631. doi: 10.1016/j.bbih.2023.100631 (PMC10209702; doi:10.1016/j.bbih.2023.100631)
Supplement: Multimedia component 1 [file mmc1.docx]

**6. Appendix A**

**Appendix Table 1:** Model fit statistics showing model fit as defined using Bayesian information criteria under different modeling assumptions.

|  | Single Class Solution | Two Classes, Both Normally Distributed | *Two Classes, One Gamma Distributed | Three Normally Distributed Classes | Three Classes, One Gamma Distributed |
| --- | --- | --- | --- | --- | --- |
| Bayesian Information Criterion | 14977.01 | 14104.67 | **14050.52** | 14116.72 | 14059.86 |
| Change in BIC |  | -872.34 | **-926.49** | -860.29 | -917.15 |

**Note:** A smaller BIC is perceived as a better overall model fit. Change in BIC reflects difference from the single class solution and, therefore, a more negative number indicates a better overall model fit.

**Appendix Table 2:** Multivariable-adjusted finite mixture model examining predictors of the class membership for heightened glial fibrillary acidic protein volume in responders in the Normal Class

|  | aOR | 95% C.I. | P value |
| --- | --- | --- | --- |
| Age, years | 1.158 | 1.097-1.221 | <0.001 |
| Female | 2.621 | 0.629-10.926 | 0.186 |
| Race/Ethnicity |  |  |  |
| Black | 3.466 | 0.995-12.073 | 0.051 |
| Other | 0.516 | 0.008-32.773 | 0.755 |
| Hispanic | 1.077 | 0.381-3.046 | 0.889 |
| Height, cm | 0.948 | 0.913-0.985 | 0.007 |
| Body Mass, kg/m^2^ | 0.911 | 0.864-0.961 | 0.001 |
| Exposure Duration, Ln-Weeks | 0.997 | 0.983-1.011 | 0.648 |
| Dust Cloud | 0.746 | 0.368-1.513 | 0.417 |
| No Supervisory Work | 0.742 | 0.376-1.464 | 0.389 |
| Post-Traumatic Stress Disorder, Symptoms | 1.059 | 0.99-1.133 | 0.096 |
| Stroke | 5.391 | 1.445-93,942.672 | 0.012 |
| Cardiovascular Disease | 1.261 | 0.748-1.000 | 0.383 |

Note: aOR= adjusted odd ratio. Estimates are significant p≤0.001.

**Appendix Table 3:** Symptom-specific predictors examining the distribution volume of glial fibrillary acidic protein, in pg/ml, in plasma

|  | Model 1 | | |  | Model 2 | | |
| --- | --- | --- | --- | --- | --- | --- | --- |
| Characteristics | B | SE | P value |  | B | SE | P value |
| Re-experiencing symptoms | -0.026 | 0.010 | 0.007 |  | -0.025 | 0.006 | <0.001 |
| Avoidance symptoms | 0.001 | 0.007 | 0.877 |  |  |  |  |
| Hyperarousal Symptoms | 0.008 | 0.008 | 0.309 |  |  |  |  |
| Negative experiences | 0.007 | 0.012 | 0.559 |  |  |  |  |
|  |  |  |  |  |  |  |  |
| Depressive Symptoms |  |  |  |  | 0.010 | 0.005 | 0.030 |
| **Note**: Models adjust for age, sex, and race/ethnicity. Estimates are significant p≤0.001. | | | | | | | |
